# The Registration of Scottish Fever Nurses

**Published:** 1921-06-18

**Authors:** Claude B. Ker

**Affiliations:** City Hospital, Comiston Road, Edinburgh


### THE EDITOR'S LETTER-BOX.

**Correspondence on all subjects is invited, but we cannot in any way be responsible for the opinions expressed by our correspondents, who must give their name and address as a guarantee of good faith, but not necessarily for publication. Correspondents are reminded that brevity of style and conciseness of statement greatly facilitate early insertion.**

### The Registration of Scottish Fever Nurses.

*To the Editor of* THE HOSPITAL.

SIR,—The difference of opinion between the Scottish Nursing Council and the Scottish Board of Health regarding the registration of certificated fever nurses raises another point of interest which I have not seen discussed. When doctors, dentists, and midwives first secured registration, I believe it to be a fact that anyone who had been in *bona-fide* practice for a certain number of years was regarded as eligible for the register, whatever their qualifications. If I am right, it would be interesting to know why this precedent has not been followed in the case of nurses, and whether Parliament, which protected these other professions, understands clearly that a quite different principle has been introduced as regards nurses' registration. Personally, my sympathies are entirely with those who desire to draw a very hard-and-fast line between general training and the various forms of special training, but is it entirely equitable to make our laws retrospective?

I may add that practically none of our own nurses are concerned. For many years they have, on leaving us, proceeded to general training. I cannot regard this as in any way a hardship, as girls start nursing so young nowadays that at the age they have completed three or four years' training they cannot expect to inspire much confidence in private nursing, and they are none the worse for a year or two in addition.—I am, Sir, yours truly,

CLAUDE B. KER, M.D.

City Hospital, Comiston Road,
Edinburgh, June 12, 1921.

[In regard to the first point as to *bona-fide* practice, Dr. Ker will find that paragraph 3 (2) (*c*) of the Nurses' Registration Act, 1919, for England and Wales provides during the period of grace (*i.e.*, two years after the Rules come into operation) for the registration of "existing nurses"—*i.e.*, persons who for at least three years prior to November 1, 1919, were *bona-fide* engaged in practice as nurses in attendance on the sick, subject to certain qualifications as to age, character, and the conditions under which the knowledge and experience of such nurses have been gained; and the Rules which are now awaiting the Minister's sanction deal exclusively with "existing nurses" who, being eligible, can register without examination. The point at issue with the Scottish Council is not the eligibility of Scottish certificated fever nurses to be registered, but as to which part of the English Register they shall be admitted to under reciprocal registration between the two countries. The English Council has formed under the Act a Supplementary Register for Fever Nurses, and the English Rules require that it is to this part of the Register that fever nurses, being eligible, shall be admitted. Scotland, however, has made the claim that they shall be admitted to the general part of the Register which is reserved for general nurses.—ED. THE HOSPITAL.]

